# Effectiveness of Traditional Chinese Medicineas as an Adjunct Therapy for Refractory Schizophrenia: A Systematic Review and Meta Analysis

**DOI:** 10.1038/s41598-018-24547-0

**Published:** 2018-04-18

**Authors:** Yan-Yan Wei, Wan-Fu Lin, Tian-Hong Zhang, Yun-Xiang Tang, Ji-Jun Wang, Mao-Feng Zhong

**Affiliations:** 1 Department of EEG Source Imaging, Shanghai Mental Health Center, Shanghai Jiao Tong University School of Medicine, Shanghai, 200030 P. R. China; 2 Department of Traditional Chinese Medicine, Changhai Hospital, Second Military Medical University, Shanghai, 200433 P. R. China; 3 Department of medical psychology, Faculty of Mental Health, Second Military Medical University, Shanghai, 200433 P. R. China; 4 Graduate School of Shanghai University of Traditional Chinese Medicine, Shanghai, 201203 P.R. China

## Abstract

Although recent studies focused on traditional Chinese medicine (TCM) for the treatment of refractory schizophrenia have reported that it may be beneficial, there is still lack of convincing evidence and critical meta-analytic work regarding its effectiveness as an adjunctive therapy. Therefore, we performed a meta-analysis to investigate the effectiveness of TCM in combination with antipsychotics for refractory schizophrenia. Fourteen articles involving 1725 patients published as of December 2016 were included which compared antipsychotic therapies to either TCM alone, or TCM as an adjunctive therapy. TCM was observed to have beneficial effects on aspects of the Positive and Negative Syndrome Scale (PANSS) including total score changes and negative score changes, as well as clinical effects estimated with PANSS or the Brief Psychiatric Rating Scale (BPRS). The changes in extrapyramidal side effects (RSESE) scores from baseline to the end of the treatment period were similar in two groups of related trials. TCM was also reported to mitigate some anti-psychotic related side-effects and overall, TCM adjuvant therapy was generally safe and well tolerated. While, the results indicated the potential utility of TCM as an alternative adjunctive therapeutic for refractory schizophrenia treatment, there remains a need for further high-quality studies.

## Introduction

Schizophrenia is a heterogeneous disorder characterized by varying degrees of positive psychotic symptoms, negative symptoms, and cognitive impairment^1^. In the clinic, patients may present with impaired social and occupational functioning associated with misattributions or delusions, hallucinations, cognitive deficits, thought disorder, negative symptoms, mood changes, and movement disorder, which results in the substantial burden of illness in schizophrenia^2^. It is a chronic, often disabling illness that affects approximately 24 million people worldwide^3^. In the 1970s, the standardized mortality rate of schizophrenia was 1.84-fold more than that of the general population, which increased to 3.20-fold in 1990s and the trend has continued into the last decade^4^. Although antipsychotic treatment such as clozapine may ameliorate schizophrenia symptoms, approximately 20% to 30% of schizophrenia patients fail to respond to pharmacotherapy despite their adherence^5^. The poor response of psychotic symptoms to single antipsychotic drug may be the most common reason for simultaneous prescription of multiple antipsychotic drugs or polypharmacy, which may aggravate the side effects associated with antipsychotic drugs^6–8^. Thus, refractory schizophrenia may impact patients’ quality of life and the burden of both their families and society, with estimated costs ranging from $2.4 billion to $23 billion for refractory schizophrenia alone^9^. Therefore, there exists a growing need to consider new and different treatment strategies, whether they are adjunctive or mono therapeutic, for treatment resistant or refractory schizophrenia patients who continue to have symptoms despite exhausting medical management options.

Traditional Chinese medicine (TCM) originates in China and encompasses characteristics of traditional Chinese philosophy and culture^10^. It has a long history being used to treat a range of mental disorders, including schizophrenia, and possesses a distinct perspective from medication techniques, emphasizing a holistic approach and treatment based on syndrome differentiation^11,12^. For refractory schizophrenia patients, TCM attempts to treat them based on the individual’s symptoms and signs, and such individualized treatment can maximize the effectiveness of Chinese medicine. Several analyses supporting the use of Chinese herbal medicine as a treatment for schizophrenia have been published, and concluding that adding Chinese herbal medicine to antipsychotic therapies may improve some outcomes in schizophrenia^13–15^. These investigations have been extended to patients with treatment refractory schizophrenia and several recent clinical trials involving the role of TCM as an adjunctive therapy have been published^16^, however, a meta-analysis of TCM in patients with refractory schizophrenia has yet to be conducted. In the present analysis, we aimed to assess the effect and safety of TCM in refractory schizophrenia treatment and to provide alternative treatment options for refractory schizophrenia patients.

## Results

### Study selection

**Figure 1:**
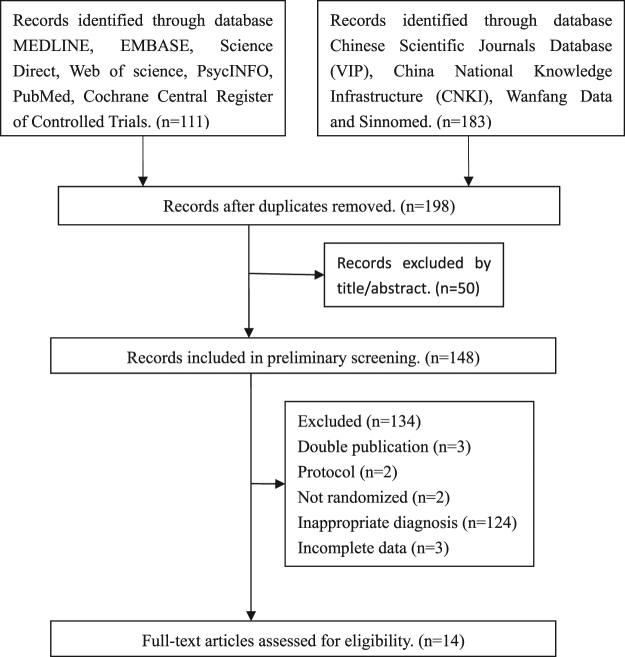
Flow diagram for process of included studies identification.

A total of 294 studies identified as potentially relevant to the research project were found in an initial search of electronic databases. After removing the duplicated records, 198 articles remained. Of these, 50 trials were excluded due to irrelevant information at the title and abstract level and a further 134 studies were excluded after an overall evaluation of the full text. The final included papers are 14 for our present meta-analysis. As showed in Fig. 1, we summarized the screening process in a flow diagram.

### Characteristics of eligible studies

The sum of enrolled participants was 1725 among the 14 included studies (868 participants in the treatment group and 857 in the control group)^17–30^, there were no observed significant differences in terms of gender, age and other demographic information. One study included Japanese patients^18^ while the remaining research subjects were all Chinese. Regarding specific selection of the treatment periods, one study reported changes after 16 weeks^25^, 8 studies were limited to 12 weeks^19,21,23,26–30^, 4 studies were limited to 8 weeks^17,20,22,24^ and one study only identified changes after 4 weeks^18^. Detailed baseline information about the participants of the included studies is presented in Table 1.

**Table 1:** Baseline information of the included studies.

Study	Method	Origin	Included (M/F)		Age (year) (M ± SD)		Mean Age of Onset Illness (year)		Duration of Illness (years)		Interventions (drug/dosage/frequency)		Outcomes measurements
			T	C	T	C	T	C	T	C	T	C	
Luo.*et al*.^25^	16-week, double-blind, parallel-group, multi-center	Inpatient	304 (248/56)	208 (170/38)	37.9 ± 10.4	36.9 ± 8.7	U	U	8.9 ± 4.7	8.4 ± 4.7	Antipsychotic (N/A); Shuxuening/40–120 mg/tid	antipsychotic (N/A); placebo/40–120 mg/tid	BPRS/SANS/RSESE
Hou.*et al*.^27^	12-week, parallel-group	Inpatient	35 (23/12)	35 (24/11)	36.9 ± 6.7	37.1 ± 6.2	U	U	6.4 ± 4.8	6.6 ± 3.7	Clozapine (N/A); promoting blood circulation formul once daily	Clozapine (N/A)	PANSS/TESS
Li *et al*.^23^	12-week, parallel-group	Inpatient	35 (23/12)	35 (24/11)	36.9 ± 6.7	37.1 ± 6.2	U	U	6.4 ± 4.8	6.6 ± 3.7	Clozapine (N/A); promoting blood circulation formul once daily	Clozapine (N/A)	PANSS/TESS
Qin *et al*.^28^	12-week, double-blind, parallel-group	Inpatient	50 (28/22)	50 (26/24)	39.7 ± 10.8	39.4 ± 10.6	U	U	9.2 ± 6.5	9.9 ± 7.8	Risperidone/4.8 ± 0.8 mg/d; Jieyu Anshen Decoction once daily	Risperidone/4.8 ± 0.8 mg/d; placebo once daily	PANSS/TESS
Zeng. *et al*.^29^	12-week, parallel-group	Inpatient	50 (22/28)	50 (24/26)	45.7 ± 15.6	45.4 ± 16.2	U	U	10.4 ± 3.5	9.9 ± 3.8	Aripiprazole/14.4 ± 5.5 mg/d; Jieyu Anshen Decoction once daily	Aripiprazole/18.6 ± 6.8 mg/d	PANSS/TESS
Chen *et al*.^17^	8-week, double-blind, parallel-group, multicenter	Outpatient, Inpatient	100 (49/51)	100 (52/48)	33.4 ± 10.5	32.5 ± 9.1	24 ± 7	24 ± 9	9 ± 3	9 ± 4	Risperidone(N/A);warm-supplement kidney yang capsule/0.9 g/tid	Risperidone(N/A);placebo capsule/0.9 g/tid	PANSS/SAPS/SANS/WCST/SDSS
Liu *et al*.^22^	8-week, parallel-group	Inpatient	56 (33/23)	52 (30/22)	36.67 ± 10.08	36.11 ± 10.52	U	U	N/A	N/A	Antipsychotic(N/A); Xuefu Zhuyu Dection once daily	Antipsychotic (N/A)	PANSS/CGI/TESS
Wang *et al*^20^	8-week, parallel-group	Inpatient	86 (52/34)	74 (45/29)	36.26 ± 12.24	31.21 ± 16.52	U	U	N/A	N/A	Antipsychotic(N/A); Shunqi Daotang Decotion or Yangxin Decotion or Xuefu Zhuyu Decotiononce daily	Antipsychotic(N/A)	PANSS/TESS
Luo. *et al*.^26^	12-week, double-blind, parallel-group	Inpatient	40 (NA)	40 (NA)	N/A	N/A	U	U	N/A	N/A	Quetiapine/345.5 ± 65.5 mg/d; Qingxintang	Quetiapine/355.5 ± 75.5 mg/d	PANSS/TESS
Yang. *et al*.^24^	8-week, parallel-group	Outpatient, Inpatient	20 (12/8)	20 (9/11)	44 ± 3.6	37.8 ± 6.2	U	U	6.8 ± 1.4	7.4 ± 2.6	Risperidon/4–6 mg/d; Shugan Jieyu Capsule/2 tablets/bid	Risperidone/4–6 mg/d	PANSS
Han. *et al*.2014^24^	12-week, parallel-group	Inpatient	60 (27/33)	60 (26/34)	46.25 ± 15.86	46.19 ± 15.22	U	U	10.73 ± 3.64	10.55 ± 3.63	Aripiprazole/15–30 mg/d; Jieyu Anshen Decoction once daily	Aripiprazole/15–30 mg/d	PANSS/TESS
Wang. *et al*.^19^	12-week, double-blind, parallel-group	Inpatient	25 (11/14)	27 (11/16)	38.44 ± 2.28	42.15 ± 2.34	U	U	7.74 ± 5.16	6.86 ± 5.59	Risperidone/6 mg/d; Eryin Jian 6 g/bid	Risperidone/6 mg/d; placebo/6 g/bid	PANSS/TESS
Wang *et al*. ^21^	12-week, double-blind, parallel-group	Inpatient	50 (26/24)	50 (25/25)	38.5 ± 10.6	38.4 ± 10.7	U	U	8.4 ± 6.3	8.5 ± 7.8	Aripiprazole;Wenyang JianpiHuoxue Decotiononce daily	Aripiprazole/15–20 mg/d	BPRS/SANS/TESS
Miyaoka *et al*.^18^	4-week, multicenter, double-blind	Inpatient	56 (34/22)	61 (39/22)	46.7 ± 9.8	46.3 ± 9.6	U	U	24.0 ± 10.4	23.6 ± 10.2	Chlorpromazine/2037.2 ± 2046.8 mg/day; yokukansan/2.5 g/tid	Chlorpromazine/1925.8 ± 2040.2 mg/day; placebo capsule/0.10 g/tid	PANSS/CGI/GAF/DIEPSS

N/A: no detailed information; U: Unclear; N: No; RSESE: rating scale for extrapyramidal side effects; CGI-S: clinical global impression severity scale; PANSS: positive and negative symptoms scale; SDSS: social disability screening schedule; WCST: Wisconsin card sorting test; TESS: treatment emergent symptoms scale; GAF: global assessment of functioning; DIEPSS: drug induced extrapyramidal symptoms scale; BPRS: brief psychiatric rating scale; SANS: scale for the assessment of negative symptoms.

### Risk of biasof included studies

**Figure 2:**
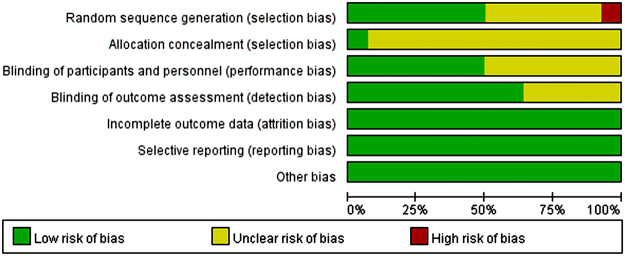
Risk of bias graph. Each risk of bias item presented as percentages across all included studies.

In order to assess the risk of bias of the included studies, Cochrane Collaboration’s risk of bias assessment tool was used, and the results are showed in Fig. 2. All these 14 trials had described the detailed stochastic methods including simple random sampling, random number table sampling, online random grouping or random number sampling. Among them, one trial described the use of sealed envelopes as a method of allocation concealment in detail^18^, while 7 studies used blind methods including single blind or double blind methods^17–19,21,25,26,28^. All included studies were parallel, and assessment before and after therapy were applied among participants. Relevant information about follow-up were reported in most studies, of which only four trials stated that there were some participants eventually left the study and the number was 50 in total among the 1725 participants^17–19,25^.

### Synthesis of results

After synthesis of results, five included trials evaluated the effectiveness of TCM alone vs. antipsychotics^17–19,25,28^, and the remaining nine compared the different effects between TCM plus antipsychotics and antipsychotics alone. As for the assessment of treatment effect, only two studies^21,25^ used brief psychiatric rating scale (BPRS) as measurement while the others used positive and negative symptoms scale (PANSS).

#### PANSS total score changes of TCM vs. antipsychotics

**Figure 3:**
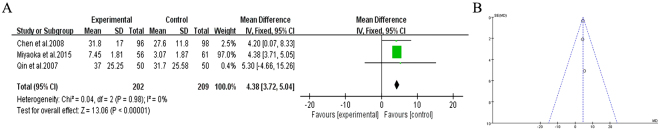
PANSS total scores of TCM vs. antipsychotics therapy. (**A**) Forest plot of comparison of the included trials. (**B**) Funnel plot of comparison of the included trials.

Three trials compared TCM vs. antipsychotics according to changes in the PANSS total scores were included in the analysis^17,18,28^. We uniformed results to the 8-week treatment effect to reduce heterogeneity. A fixed effects model was used for statistical analysis since these trials showed heterogeneity in the consistency of the trial results (χ^2^ = 0.04, *P* = 0.98, *I*^2^ = 0%). The results showed that, compared with the antipsychotics group, TCM improved the PANSS total score significantly (MD = 4.38 [3.72, 5.04]; *Z* = 13.06, *P* < 0.00001). The funnel plot was roughly symmetric (Fig. 3).

#### Negative score changes of TCM vs. antipsychotics

Four trials evaluated negative symptoms with or without TCM therapy. Heterogeneity was huge (*I*^2^ = 89%). Consequently, we performed a systemic analysis. Negative scores of patients in the TCM therapy group were reported to decrease more than those antipsychotics group, suggesting that negative symptoms may be improved through TCM therapy.

#### Clinical effects of TCM vs. antipsychotics

**Figure 4:**
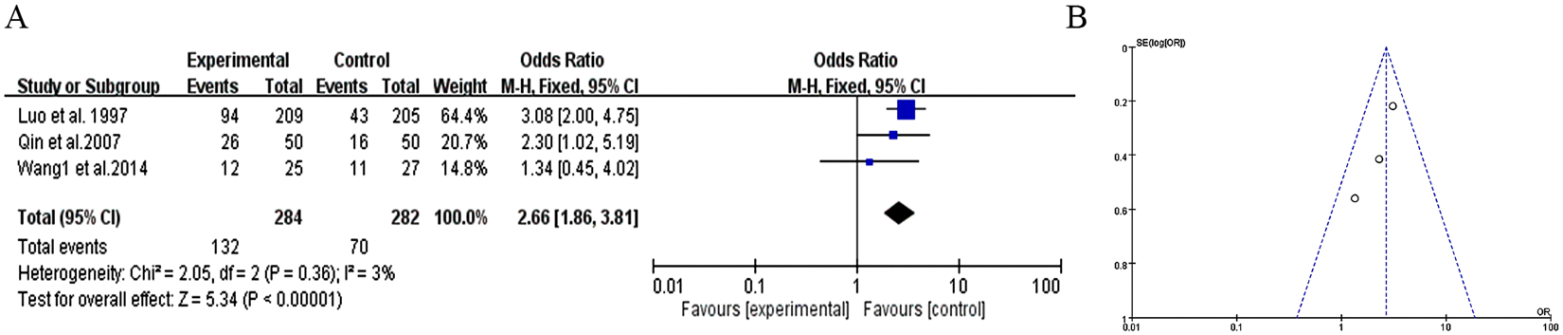
Clinical effects of TCM vs. antipsychotics therapy. (**A**) Forest plot of comparison of the included trials. (**B**) Funnel plot of comparison of the included trials.

Three trials compared the effect of TCM versus antipsychotics therapy according to clinical outcomes^19,25,28^. PANSS or BPRS scores were used for measuring the clinical outcomes, when score reduction rate ≥75% it means for the recovery, and 50–75% for significant progress, 25–50% for progress while <25% was considered negligible. Recovery and significant progress cases were accepted as clinical effects group. The three trials showed heterogeneity in the results (χ^2^ = 2.05, *P* = 0.36, *I*^2^ = 3%). Therefore, we used a fixed effects model for statistical analysis. Compared to antipsychotics treatment, TCM therapy may significantly improve the clinical effects (MD = 2.66, [1.86, 3.81]; *Z* = 5.34, *P* < 0.00001), suggesting that TCM therapy contribute to improving clinical effects of treatment in patients with refractory schizophrenia (Fig. 4).

#### PANSS total score changes of TCM plus antipsychotics vs. antipsychotics alone

**Figure 5:**
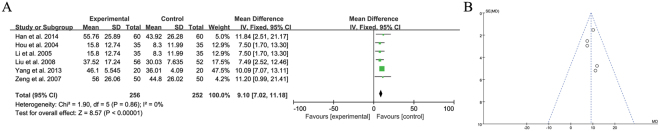
PANSS total score of TCM plus antipsychotics vs. antipsychotics therapy alone. (**A**) Forest plot of comparison of the included trials. (**B**) Funnel plot of comparison of the included trials.

Eight trials compared the effect of TCM combined with antipsychotics therapy versus antipsychotics therapy alone according to changes in the PANSS total score^20,22–24,26,27,29,30^. Analysis showed the significant heterogeneity in the consistency of the trial results (χ^2^ = 16.21, *P* = 0.02; *I*^2^ = 57%), while the benefit effect was significant. Further analysis showed that study of Wang *et al*.^20^ used Shunqi Daotang Decotion or Yangxin Decotion or Xuefu Zhuyu Decotiononce daily rather than a single decotion and the study of Wang *et al*.^26^ did not report the years of patients in different groups, which were observed with extreme heterogeneity. After they were excluded, no heterogeneity was observed. Therefore, a fixed effects model was used for statistical analysis. Analysis results showed that TCM as an adjuvant therapy may have a significant beneficial effect compared to antipsychotics therapy alone in improving the PANSS total score (MD = 9.1[7.02,11.18]; *Z* = 8.57, *P* < 0.00001) (Fig. 5).

#### Negative score of TCM plus antipsychotics vs. antipsychotics alone

**Figure 6:**
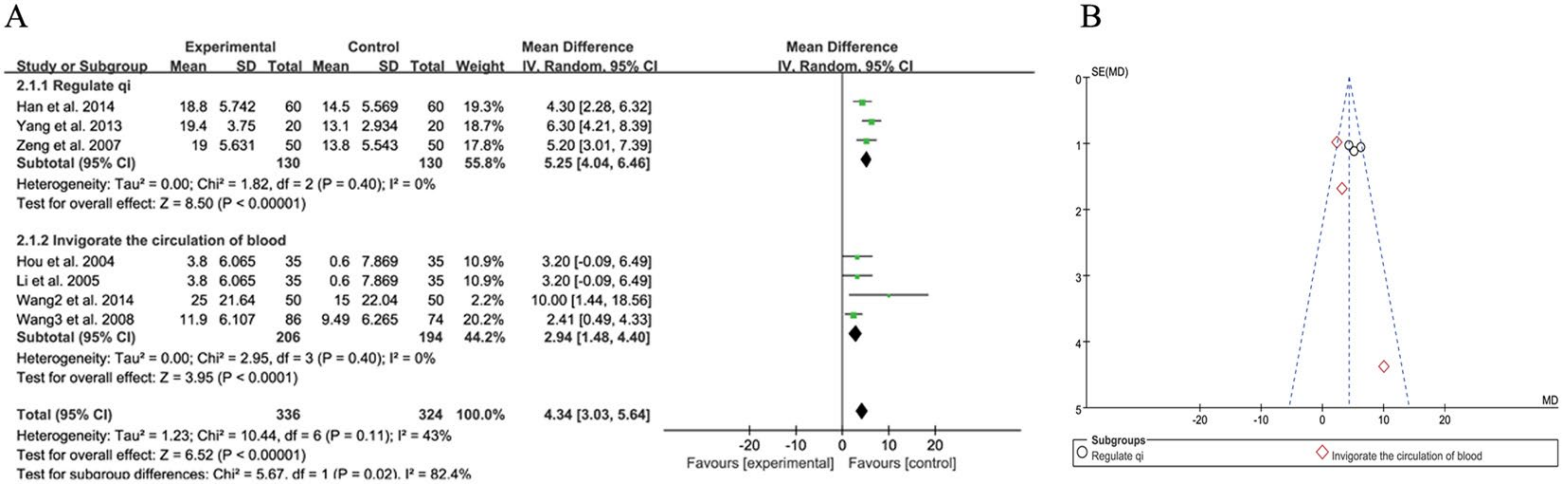
Negative score of TCM plus antipsychotics vs. antipsychotics therapy alone. (**A**) Forest plot of comparison of the included trials. (**B**) Funnel plot of comparison of the included trials.

Eight trials compared the effect of TCM combined with antipsychotics therapy versus antipsychotics therapy alone through the changes in the PANSS negative score or SANS score^20,21,23,24,26,27,29,30^. The results showed significant heterogeneity in the consistency of the trial results (χ^2^ = 24.00, *P* = 0.001; *I*^2^ = 71%). Further analysis showed that removal of the one most extreme study^26^ may decrease the heterogeneity in the consistency of the trial results (χ^2^ = 10.44, *P* = 0.11; *I*^2^ = 43%) and thus a random effects model was used for statistical analysis. Meanwhile, a subgroup analysis base on the efficacy of different Chinese medicine decoction or herb mix was necessary to estimate the evidence of heterogeneity. Since the included trials all functioned as ‘regulate qi’ and ‘invigorate the circulation of blood’, we applied the subgroup analysis and the result showed that heterogeneity was associated with different efficacy such as ‘regulate qi’ and ‘invigorate the circulation of blood’. The finally results also showed a significant beneficial effect of TCM as an adjuvant was observed when compared to antipsychotics therapy alone in improving the negative score (MD = 4.34 [3.03, 5.64]; *Z* = 6.52, *P* < 0.00001) (Fig. 6).

#### Clinical effects of TCM plus antipsychotics vs. antipsychotics alone

**Figure 7:**
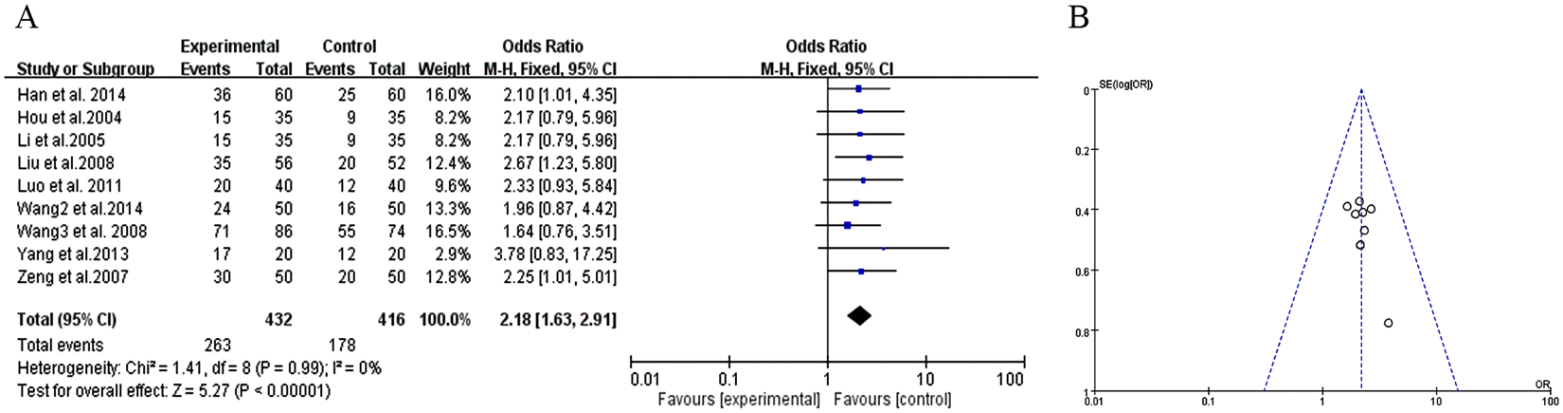
Clinical effects of TCM plus antipsychotics vs. antipsychotics therapy alone. (**A**) Forest plot of comparison of the included trials. (**B**) Funnel plot of comparison of the included trials.

Nine trials compared the effect of TCM combined with antipsychotics versus antipsychotics therapy alone through the clinical effects^20–24,26,27,29,30^. They showed heterogeneity in the results (χ^2^ = 1.41, *P* = 0.99; *I*^2^ = 0%). Therefore, a fixed effects model was used for statistical analysis. Compared to antipsychotics therapy alone, TCM adjuvant therapy significantly improved the clinical effects (MD = 2.18, [1.63, 2.91]; *Z* = 5.27, *P* < 0.00001), indicating that TCM as an adjuvant therapy may contribute to improve clinical effectiveness of treatment in refractory schizophrenia patients (Fig. 7).

### Side effects

Although there were five trials involving TESS scores and the result showed non-significant differences between TCM and antipsychotics treatment (Table 2), TCM treatment appeared to alleviate some side effects in the studies. As shown in Table 3, the result indicated that compared with antipsychotics treatment, TCM may reduce tremor and constipation events significantly (*I*^2^ = 0%, *Z* = 2.81, *P* = 0.005 and *I*^2^ = 66%, *Z* = 3.44, *P* = 0.0006, respectively). However, it seemed that insomnia may not be improved in TCM treatment group (*I*^2^ = 14%, *Z* = 1.31, *P* = 0.19).

**Table 2:** TESS scores after treatment.

Author (year)	Experimental			Control		
	Mean	SD	Total	Mean	SD	Total
Han *et al*.^30^	4.86	2.43	60	4.81	2.52	60
Qin *et al*.^28^	4.9	2.4	50	4.7	2.6	50
Wang *et al*.^21^	1.8	2.5	50	1.7	2.5	50
Wang *et al*.^20^	3.95	2.36	86	3.97	2.29	74
Zeng *et al*.^29^	4.9	2.4	50	4.7	2.6	50

**Table 3:** Side effects of TCM plus antipsychotics vs. antipsychotics therapy alone.

Adverse events	No. of studies	TCM/antipsychotics (n/n)	RR/OR [95% CI]
Tremor	5^22–24,27,29^	196/192	0.40 [0.21, 0.76]*
Constipation	5^22–24,27,29^	196/192	0.16 [0.06, 0.45]*
Insomnia	7^17,18,22–24,27,29^	348/351	1.47 [0.77, 2.78]

^*^P < 0.05.

### Tolerance

Interestingly, there were two trials which reported both TCM and antipsychotic groups improving in terms of RSESE scores, a measure of extrapyramidal side effects^17,25^. However, these results showed that changes in scores from baseline to end point on the RSESE were not significantly different between TCM and antipsychotics therapy groups (MD = 0.14, [−0.22, 0.51]; *Z* = 0.76, *P* = 0.45).

### Cognitive function

Among the including 14 studies, there only one trial evaluated TCM treatment versus antipsychotics therapy in terms of cognition via the Wisconsin Card Sorting Test (WCST) scores^17^. In this study, the WCST scores were similar in the 2 groups at baseline. However, at the end point, the TCM group had improved significantly at the end point in all categories such as the number of completed categories, perseverative responses and total number of errors when compared to the antipsychotics therapy group. (*P* < 0.01).

## Discussion

While, clozapine is presently considered as the first choice for symptomatic treatment of refractory schizophrenia. Under conditions of continued poor treatment response and high relapse rates, psychiatrists often attempt to combine clozapine with other antipsychotics rather than continue monotherapy with a single drug. In its attempt to generate individualized treatments, TCM’s focus on the concept of holistic practice and goal to treat patients as a whole rather than just the disease, shows substantial promise to improve clinical outcomes. Numerous studies have suggested the existence of therapeutic benefits of TCM for persistent negative symptoms, cognitive impairment, and adverse side effects in schizophrenic patients^31^. It also could alleviate hyperprolactinemia in schizophrenic patients too^32^. However, it is also commonly observed that herbal medicines are accompanied by their own risks, side-effect profile, and in some cases may inadvertently induce psychosis in schizophrenia along with unknown herb-drug interactions^33^. In order to summarize the evidence about the effects of TCM as an adjunct therapy for refractory schizophrenia treatment, we performed the present systematic review and meta-analysis. Our results supports evidence that TCM can help improve refractory schizophrenia symptoms and in some cases may serve to mitigate common side-effects of antipsychotic medicines. Due to the small sample sizes of the selected studies and the limitations of study designs, it is insufficient of the current evidence to make a routine recommendation of TCM for refractory schizophrenia treatment. However, the existence of clinical improvements as evidenced by the PANSS scores under TCM as adjunct therapy suggests that further research, particularly well-controlled studies are merited. Significant heterogeneity was observed for two comparisons, which was solved by subgroup analysis. Among the Chinese medicines adopted in the studies, the results of the statistical meta-analysis suggested that Xuefu Zhuyu Dection, Jieyu Anshen Decoction, Shugan Jieyu Capsule, Shunqi Daotang Decotion added to antipsychotics may significantly improve on PANSS total scores and clinical effects compared with antipsychotics treatment alone in these patients. A similar outcome was noted in the studies which examined TCM alone vs. antipsychotics. Regarding the impact on negative symptomology, a proper meta-analysis through subgroup conducted by ‘regulate qi’ and ‘invigorate the circulation of blood’ showed the changes in negative scores or SANS from baseline to endpoint of the patients in TCM group indicated that TCM may have improvement effects over antipsychotics exclusively.

Additionally, TCM was reported to reduce side effects such as tremor and constipation, however other studies found no difference. The impact of TCM on cognitive deficits is unclear as only one study attempted to investigate this via the WCST.

Although our present study indicated an apparent positive role of TCM as adjuvant therapy for refractory schizophrenia patients, it is still premature to conclude the overall safety and efficacy of TCMs under this patient context. There are several limitations in this study. First of all, the strength of this meta-analysis was impacted by the relatively limited availability of relevant studies in part due to the specific patient conditions being investigated, and limited use of TCM outside of Chinese populations. Therefore, in order to make our conclusions more objective and substantial, more high-quality trials with larger numbers of subjects are required. Secondly, the quality of methodologies used in the presently reported studies was somewhat poor. In spite that all included studies demonstrated randomization, there were still six trails only noted that stochastic grouping methods were used without more details. Only one study provided information about using sealed envelopes as allocation concealment and seven studies did not use the blinding method that selection bias may increase in the remaining studies since subjective factors of researchers that tend to assign specific patients into the control or treatment groups. Furthermore, there were six studies failed to mention information regarding dropouts, which might exaggerate the therapy effects. Finally, although TCM may act as a beneficial adjuvant therapy, details about the category, the effects of specific herb medicine and dosages were absent in most of the studies included in this analysis. Both antipsychotic drugs and TCM treatments are subject to high degrees of patient variability and may feature yet-undescribed treatment interactions which may confound observed treatment effects and side-effect profiles. Although opportunities may exist to select specific comparative therapeutic regimes in individual studies in order to control for such variability, the scarcity of quality TCM research in relation to antipsychotics treatment, especially under the context of treatment-refractory schizophrenia, introduces substantial difficulty in drawing meta-analytic conclusions without further investigation. Therefore, while promising, there exists a strong need for additional research, particularly with studies that demonstrate high-quality experimental practice and reporting of patient selection, information, drop-out, and clinical changes.

## Conclusions

In spite of the limitations of the small sample sizes and design methods of the selected studies and the limitations of study designs, this meta-analysis provides a consolidation of evidence regarding the potential advantages of TCM as an alternative medicine that is suitable for refractory schizophrenia patients. However, more well-designed studies are needed to further clarify thus effects of TCM on refractory schizophrenia patients.

## Methods

### Data sources and search strategies

For our review, we identified randomized, controlled clinical trials that compared the effectiveness of antipsychotics alone and antipsychotics with TCM as adjunctive treatment in refractory schizophrenia through a comprehensive, systematic literature search in MEDLINE, EMBASE, Science Direct, Web of science, PsycINFO, PubMed, Cochrane Central Register of Controlled Trials, Chinese Scientific Journals Database (VIP), China National Knowledge Infrastructure (CNKI), Wanfang Data and Sinnomed. All the above electronic databases were searched from their inception until 17 January 2017. In order to collect a more comprehensive data, the search strategies for all studies containing the following terms of “schizophren*”, “Schizophrenia*”, “Schizophrenic Disorder” or “Disorder, Schizophrenic” and “medicine, Chinese traditional” or “Chinese herbs”. In Chinese databases, we used “”,“” or “, ” and “” or “”. There were no language, date, document type or publication status limitations for the inclusion of records, thus reducing the risk of publication bias. Through assessing the searched studies by reading the title and abstract, we finally extracted the studies concerning antipsychotics alone or with TCM in treating refractory schizophrenia.

### Study selection

Our inclusion criteria were as follows: (1) Types of studies: Randomized controlled trials (RCTs); (2) Diagnoses standards: Diagnoses included schizophrenia, schizoaffective disorder or schizophreniform disorder. Participants had to have demonstrated a resistance to treatment as criteria used by Kay *et al*.^34^. These criteria included patients with a length of at least 3 years’ history of documented treatment-resistant status, which is defined as the absence of clinically significant improvement after treatment with at least two neuroleptics for 6 weeks or longer after receiving a full dose equivalent to 600 mg/day of chlorpromazine, presence of persistent positive symptoms as evidenced by a score of at least 10 on the positive symptom subscale of the Positive and Negative Syndrome Scale (PANSS); (3) Types of interventions: TCM, conventional antipsychotics medicine or their combination were included if detailed data were available. Patients with antipsychotics treatment were included as control groups; (4) Outcome measures: The primary outcome was the mean change from baseline to end point in overall symptoms of schizophrenia as measured by PANSS and the Brief Psychiatric Rating Scale (BPRS)^34,35^. Any other validated scale for the assessment of overall schizophrenia symptoms such as Treatment Emergent Symptom Scale (TESS) might also be included. Our exclusion criteria were as follows: (1) Review or animal experiments trails; (2) Comparisons between different types of TCMs. There was extensive crossover between the TCM and control groups; (3) The control group was not treated with antipsychotics, or there was no control groups, or poor between-group baseline; (4) Data on clinical trials was deficiency.

### Data extraction and analysis

Data were extracted by 2 reviewers (YYW and MFZ), any discrepancies were resolved by the third author (WFL). For more details about the selected studies, the following data was extracted: the information of authors such as name and affiliation, year of publication, research time and location, study sample size, patients’ age, gender, diagnostic criteria, outcome measures, treatment intervention regimes, side effects, effective rate and results.

### Quality appraisal

The quality appraise and risk of bias of each included study was independently evaluated by two reviewers with Cochrane Collaboration’s risk of bias assessment tool was applied. The tool concerns the study randomization method, concealment of treatment allocation, blinding of patients, researchers and assessors (intervention, data collection and analysis), outcome measures such as completeness of outcome data and follow-up, and any other potential sources of bias. Finally, a consensus about the methodological quality of all the studies was achieve since any disagreement between the two reviewers will be resolved through discussion.

### Data synthesis and statistical methods

We used RevMan 5.2 software for the meta-analyses. Weighted mean difference (WMD) with 95% confidence intervals (CI) was calculated for continuous data. Heterogeneity was tested using a standard chi-square test and *I*^2^ statistic, which does not depend on the number of studies in the meta-analysis and hence has greater power to detect heterogeneity when the number of studies is small. Based on the heterogeneity of different trials, fixed-effect model or random-effect model would be used for further analysis. Two-tailed *P* values less than 0.05 were considered statistically significant. Funnel plot analysis was used to detect Publication Bias.

## References

[1] Mikell CB, Sinha S, Sheth SA (2016). Neurosurgery for schizophrenia: an update on pathophysiology and a novel therapeutic target. Journal of neurosurgery.

[2] Kim, Y. K., Choi, J. & Park, S. C. A Novel Bio-Psychosocial-Behavioral Treatment Model in Schizophrenia. *International journal of molecular sciences* **18**, 10.3390/ijms18040734 (2017). 10.3390/ijms18040734 PMC5412320 28358303

[3] Abi-Dargham A (2014). Schizophrenia: overview and dopamine dysfunction. The Journal of clinical psychiatry.

[4] Catts SV, O’Toole BI (2016). The treatment of schizophrenia: Can we raise the standard of care?. The Australian and New Zealand journal of psychiatry.

[5] Li XB, Tang YL, Wang CY, de Leon J (2015). Clozapine for treatment-resistant bipolar disorder: a systematic review. Bipolar disorders.

[6] Takeuchi H, Fervaha G, Remington G (2016). Incidence of antipsychotic-associated side effects: Impact of clinician versus patient ratings and change versus absolute scores. Journal of clinical psychopharmacology.

[7] canavan2016.pdf. 10.1080/00207411.2016.1215217.

[8] Fakhoury M (2016). Could cannabidiol be used as an alternative to antipsychotics?. Journal of psychiatric research.

[9] Van Sant SP, Buckley PF (2011). Pharmacotherapy for treatment-refractory schizophrenia. Expert opinion on pharmacotherapy.

[10] Deng, H. & Adams, C. E. Traditional Chinese medicine for schizophrenia: A survey of randomized trials. *Asia-Pacific psychiatry: official journal of the Pacific Rim College of Psychiatrists* **9**10.1111/appy.12265 (2017). 10.1111/appy.12265 27734592

[11] Chen ZG, Luo H, Xu S, Yang Y, Wang SC (2015). Study on the methodology of developing evidence-based clinical practice guidelines of Chinese medicine. Chinese journal of integrative medicine.

[12] Zhai XF, Ling CQ (2012). Diagnosis and treatment of malignant tumors using integrated traditional and western medicine: progress, challenges and reflections. Chinese journal of integrative medicine.

[13] Rathbone J (2007). Chinese herbal medicine for schizophrenia: cochrane systematic review of randomised trials. The British journal of psychiatry: the journal of mental science.

[14] Werneke U (2008). Review: adding Chinese herbal medicine to antipsychotics may improve some outcomes in schizophrenia, but more high quality trials are needed. Evidence-based mental health.

[15] Singh V, Singh SP, Chan K (2010). Review and meta-analysis of usage of ginkgo as an adjunct therapy in chronic schizophrenia. The international journal of neuropsychopharmacology.

[16] Miyaoka T (2009). Yi-gan san as adjunctive therapy for treatment-resistant schizophrenia: an open-label study. Clinical neuropharmacology.

[17] Chen ZH (2008). Effects of warm-supplementing kidney yang (WSKY) capsule added on risperidone on cognition in chronic schizophrenic patients: a randomized, double-blind, placebo-controlled, multi-center clinical trial. Human psychopharmacology.

[18] Miyaoka T (2015). Efficacy and safety of yokukansan in treatment-resistant schizophrenia: a randomized, double-blind, placebo-controlled trial (a Positive and Negative Syndrome Scale, five-factor analysis). Psychopharmacology.

[19] Wang D, Jia HX (2014). Effect of Qingreyangyin on MDA and SOD in peripheral blood of refractory schizophrenia. Journal of Shandong University of Traditional Chinese Medicine.

[20] Wang JX (2008). The research on the treatment of the chronic schizophrenia by the chinese and western medicine. Chinese Journal of Practical Traditional Chinese with Western Medicine.

[21] Wang X, Gong Q, Wu AM (2014). Clinical study on treatment of negative symptoms of chronic psychotic schizophrenia by combination of Wenyang Jianpi Huoxue decoction and aripiprazole. Journal of Sichuan of Traditional Chinese Medicine.

[22] Liu B, Wang JX (2008). Clinical observation on treating refractory schizophrenia by thetherapy of Huoxue Huayu. Chinese Joumal of the Practical Chinese with Modern Medicine.

[23] Li YE, Guo XL, Sun JP (2005). Treatment of refractory schizophrenia by integrated traditional Chinese and Western medicine. Journal of Nursing Science.

[24] Yang RK (2013). Clinical efficacy observation on treating schizophrenia with Shugan Jieyu capsule and risperidone. Clinical Journal of Traditional Chinese Medicine.

[25] Luo HC (1997). Therapeutic effect of Shuxuening combining neuroleptic for the treatment of chronic schizophrenia—A double blind study. Chinese Journal of Integrated Traditional and Western Medicine.

[26] Luo JW, Zeng DZ, Zhou GM (2011). Auxiliary effects of Qingxintang in treatment of the chronic schizophrenia. Journal of Hainan Medical College.

[27] Hou XX, Wang LJ, Liu H, Lin SM (2004). A comparative study of the treatment of refractory schizophrenia with Huoxue Huayu combined with clozapine. Chinese Journal of Clinical Medicine.

[28] Qin TX, Guo XX, Zeng DZ, Fan XW (2007). Therapeutic effect and safety of Jieyu Anshen broth combining to Risperidone in treatment of the chronic schizophrenia. Modern Journal of Integrated Traditional Chinese and Western Medicine.

[29] Zeng DZ, Luo JW, Fan XW, Zhong YF, Liu ZS (2007). Clinical observation on effect of Jieyu Anshen decoction combined with aripiprazole in treating chronic schizophrenia. Chinese Journal of Integrated Traditional and Western Medicine.

[30] Han WD, Li L, Jiang R, Liu HB (2014). Efficacy and safety of Jieyu Anshen decoction combined with aripiprazole tablets in treating chronic schizophrenia. Modern Journal of Integrated Traditional Chinese and Western Medicine.

[31] Zhang ZJ (2011). An epidemiological study of concomitant use of Chinese medicine and antipsychotics in schizophrenic patients: implication for herb-drug interaction. PloS one.

[32] Man SC (2016). Peony-Glycyrrhiza Decoction for Antipsychotic-Related Hyperprolactinemia in Women With Schizophrenia: A Randomized Controlled Trial. Journal of clinical psychopharmacology.

[33] Peterson E, Stoebner A, Weatherill J, Kutscher E (2008). Case of acute psychosis from herbal supplements. South Dakota medicine: the journal of the South Dakota State Medical Association.

[34] Kay SR, Fiszbein A, Opler LA (1987). The positive and negative syndrome scale (PANSS) for schizophrenia. Schizophrenia bulletin.

[35] Overall JE, Gorham DR (1962). The Brief Psychiatric Rating Scale. Psychological Reports.

